# Audiometric Profile in Post-COVID-19 Patients

**DOI:** 10.1055/a-2873-4569

**Published:** 2026-08-14

**Authors:** Mathias Antonialli Castoldi, Fernando de Andrade Quintanilha Ribeiro

**Affiliations:** 1 Department of Otorhinolaryngology, Faculdade de Ciências Médicas da Santa Casa de São Paulo, São Paulo, Brazil; 2 Department of Otolaryngology, Faculdade de Ciências Médicas da Santa Casa de São Paulo, São Paulo, Brazil

**Keywords:** COVID-19, hearing loss, tinnitus, tonal thresholds

## Abstract

**Introduction:**

There is uncertainty about whether COVID-19 is a risk factor for hearing loss.

**Objective:**

To evaluate the audiometric profile of patients post-COVID-19 infection and compare it to unaffected individuals, clarifying the correlation between COVID-19 and hearing loss. Additionally, we sought correlations between reported otorhinolaryngological symptoms during COVID-19 and changes in tonal thresholds.

**Methods:**

A cross-sectional study compared the pure-tone thresholds of 120 individuals with a confirmed history of COVID-19 and 120 unexposed controls, evaluated between 2021 and 2023.

**Results:**

The median tonal thresholds of exposed individuals were predominantly higher than those of unexposed individuals in both ears. The rate of hearing threshold alteration was 28.3% in exposed individuals versus 15% in unexposed individuals, with an odds ratio (OR) of 2.24 (95% CI: 1.18–4.25). Comparison of thresholds between the right and left ears of each exposed individual demonstrated asymmetric hearing impairment. The distribution of otorhinolaryngological symptoms included: nasal symptoms 67.5%; cough 65.8%; loss of smell 60%; loss of taste 56.7%; sore throat 47.5%; dizziness 30.8%; hoarseness 21.7%; tinnitus 19.2%; earache 18.3%; hearing loss 18.3%; and facial paralysis 0.8%. The association between reported symptoms and changes in pure-tone thresholds was statistically significant only for hearing loss (p < 0.001; OR = 8.9) and tinnitus (p = 0.005; OR = 3.7).

**Conclusion:**

Individuals exposed to COVID-19 showed worse tonal thresholds and a higher rate of hearing changes compared with non-exposed individuals.

## Introduction

A novel coronavirus that emerged in Wuhan, China, in December 2019 was designated Severe Acute Respiratory Syndrome Coronavirus 2 (SARS-CoV-2) and rapidly spread worldwide. 1 2 3 The disease caused by Sars-CoV-2, COVID-19, was characterized by the World Health Organization as a pandemic. SARS-CoV-2 is a highly pathogenic single-stranded RNA virus that enters host cells via the angiotensin-converting enzyme 2 (ACE2) receptor, and can cause an uncontrolled increase in the release of cytokines, leading to a drastic recruitment of leukocytes in various organs. COVID-19 can manifest with symptoms ranging from mild to very severe, with ENT symptoms being common among infected patients. 4 5 6

ENT evaluation may reveal cough, dyspnea, sore throat, rhinorrhea, nasal congestion, enlarged cervical lymph nodes, tonsillar edema, dizziness, hyposmia/anosmia, and dysgeusia, 7 8 in addition to reports of a symptom which became the focus of this study: hearing loss — an atypical manifestation that may be part of the symptom spectrum of COVID-19. 9

Several studies on the association between hearing loss and COVID-19 are emerging in the literature, but there is disagreement and inconsistency between the results obtained in different countries. There is a low level of evidence due to the lack of studies with a control group, high heterogeneity of the studies reviewed, uncertainties in data collection, lack of studies with larger populations and long-term follow-up. 10 11 12 13 14 15 16

Therefore, the correlation between COVID-19 and hearing loss has not yet been fully clarified. The present study aims to contribute to clarifying this hypothesis by evaluating the audiometric profile of individuals infected with SARS-CoV-2, addressing methodological limitations identified in previous research on this topic.

## Methods

This study had a sample size of 240 individuals, evaluated between 2021 and 2023. The COVID-19-exposed group consisted of 120 individuals (91 women [75.8%] and 29 men [24.2%]) who presented to the general ENT outpatient clinic. Inclusion criteria for the exposed group were: age between 18 and 50 years; confirmed SARS-CoV-2 infection by RT-PCR; and voluntary agreement to participate, documented by signed informed consent. Patients presenting comorbidities with audiovestibular repercussions, **risk of noise-induced hearing loss** and otological symptoms before COVID-19 were excluded from the study.

The unexposed group comprised 120 individuals (60 women and 60 men [50% each]) identified by reviewing electronic medical records at an occupational health clinic. Only audiometric data from individuals who had not reported a COVID-19 diagnosis or symptoms at the time of audiometry were included . Individuals who carried out periodic and dismissal audiometries, which could suggest a noise-induced hearing loss (NIHL) process, were excluded.

For the group of exposed individuals, the researcher, during outpatient care, selected patients who had a positive history for COVID-19 confirmed by RT-PCR, and carried out a detailed clinical history and otoscopy to exclude patients with otological diseases pre-dating COVID-19 , as well as previous comorbidities that may have cochlear and vestibular repercussions. A purpose-designed symptom questionnaire addressing the following variables was then administered : name; age; sex; type of treatment undergone: home treatment, hospitalization in a ward or Intensive Care Unit (ICU); ENT symptoms (nasal symptoms [sneezing, runny nose and nasal obstruction/congestion], sore throat, loss of smell, loss of taste, cough, hoarseness, earache, dizziness, tinnitus, hearing loss and peripheral facial paralysis) and time of the diagnosis of COVID-19 (less than 3 months, between 3 and 6 months or greater than 6 months). These patients underwent a tonal audiometry, performed by speech-language pathologists using the Interacoustics AD629 audiometer in a calibrated acoustic booth (ISO 8253-1 standard). . 17

For the non-exposure group, electronic medical records from the Occupational Health and Safety Software® system were reviewed . First, we filtered the medical records of individuals evaluated between 2021 and 2023. Using the medical records available, we identified individuals infected with COVID-19 confirmed by RT-PCR who required time off work. Those who showed no symptoms or tested negative in periodic RT-PCR screening were candidates for the unexposed group. From the medical records obtained, we selected those aged between 18 and 50 years, who had undergone a pre-employment audiometry, until we reached 120 patients. The audiometries were performed by phonoaudiologists, using an Interacoustics AD229 audiometer, also calibrated according to the technical standard ISO 8253-1, 17 in an acoustic booth. Comorbidities and sex distribution (achieved equally) were left to chance during participant selection to ensure the group was representative of the general population.

The material was tabulated in the Microsoft Excel® program to create the database. Then, Data distribution was assessed using the Shapiro-Wilk test, which yielded p < 0.05, confirming a non-normal distribution. The Mann-Whitney test was subsequently used for between-group comparisons. Additionally, the presence of outlying values was observed in the data obtained. Therefore, we are faced with a non-parametric distribution and adopted Median values in our analysis to compare tonal thresholds between groups. The chi-square test was used to compare rates of hearing threshold alteration between groups. The chi-square test or Fisher's exact test, with odds ratios (ORs), was used to assess associations between reported ENT symptoms and audiometric changes. The Wilcoxon signed-rank test was used to compare thresholds between the right and left ears within the exposed group.

## Results

**Fig. 1:**
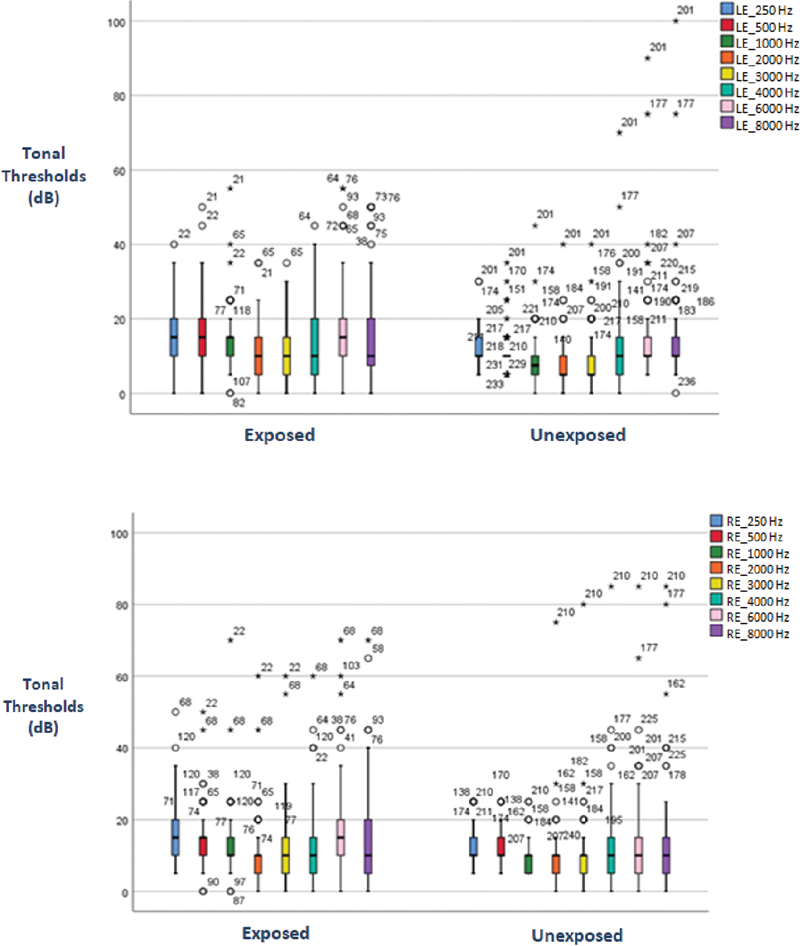
OD, right ear; OE, left ear. Asterisks (*) and circles (o) indicate outlier values; numbers correspond to the positions of outliers in the database.

The medians of the tonal thresholds were calculated for each auditory frequency studied and, those of exposed individuals were predominantly higher. The data are presented as box plots. These median values are shown for the right and left ears of both groups, along with outlier values representing atypical and extreme thresholds ( Fig. 1 ).

The Chi-square test was used to calculate the frequency of altered hearing thresholds below 25 dB. In the exposed group, this rate was 28.3%, compared with 15% in the unexposed group (p = 0.012). The OR was 2.24 (95% CI: 1.18–4.25), indicating approximately twice the risk of hearing threshold alteration in exposed compared with unexposed individuals.

The Wilcoxon signed-rank test was used to compare right and left ear thresholds within the exposed group. Higher thresholds were observed in the right ear at 500 Hz (p = 0.02) and in the left ear at 4,000 Hz (p = 0.051), 6,000 Hz (p = 0.055), and 8,000 Hz (p = 0.02). No statistically significant differences were observed at 250 Hz (p = 0.08), 1,000 Hz (p = 0.327), 2,000 Hz (p = 0.793), or 3,000 Hz (p = 0.377).

Among exposed participants, the reported ENT symptom frequencies were as follows: nasal symptoms, 67.5%; cough, 65.8%; loss of smell, 60.0%; loss of taste, 56.7%; sore throat, 47.5%; dizziness, 30.8%; hoarseness, 21.7%; tinnitus, 19.2%; earache, 18.3%; hearing loss, 18.3%; and facial paralysis, 0.8%.

To evaluate the association between these reported ENT symptoms with the audiometric changes recorded in these participants, the Chi-square or Fisher's Exact test was used. A statistically significant association was found only for the complaints of hearing loss (p < 0.001) and tinnitus (p = 0.005). For tinnitus, an OR of 3.7 (95% CI: 1.4–9.6) was obtained, and for hearing loss, an OR of 8.9 (95% CI: 3.2–24.9). In other words, participants with SARS-CoV-2 who reported hearing loss had 8.9 times the odds of altered pure-tone thresholds, and those who reported tinnitus had 3.7 times the odds, compared with those who did not experience these symptoms.

In the sample of those exposed to COVID-19, there was a predominance of women (75.8%) compared to men (24.2%), unlike the non-exposed group, which had a distribution of 50% for men and women. The median age of the group of participants who had COVID-19 was 34.5 years (SD= 8.6) and of those who did not was 32 years (SD = 8.1). Only sensorineural hearing loss (SNHL) was identified in the exposed group, whether unilateral or bilateral.

At follow-up, 62.5% of patients underwent audiometry between six months and two years after COVID-19 diagnosis; 19.2% between three and six months, and 18.3% in less than three months after the disease. With regard to where individuals were treated during COVID-19, we obtained the following distribution: 92.5% underwent home treatment; 5.8% treatment in a hospital ward and 1.7% treatment in an U.

## Discussion

The use of self-reported hearing assessment in previous studies, instead of objective audiometric testing, has resulted in inaccurate estimates of COVID-19-related hearing loss. 11 12 13 14 15 Therefore, pure-tone audiometry was conducted with all participants by speech-language pathologists in calibrated acoustic booths using audiometers calibrated to the same technical standard (ISO 8253-1), minimizing instrument bias.

The non-systematization of data collection by previous research did not ensure the absence of biases, such as: hearing loss prior to COVID-19 or cochleovestibular symptoms that were not caused by Sars-Cov-2. 10 11 12 13 14 15 Therefore, the criteria used in the selection of participants exposed in this study were crucial to avoid such limitations. These were: confirmed COVID-19 diagnosis by RT-PCR (the gold-standard diagnostic method); exclusion of individuals over 50 years of age (to minimize presbycusis); and exclusion of individuals with pre-existing symptoms or comorbidities affecting cochlear or vestibular function.

For the unexposed group, pre-employment audiometric tests of individuals who had not had a diagnosis or symptoms of COVID-19 by the time of the hearing examination were used. Individuals who had had dismissal or periodic audiometries were excluded, thereby decreasing the possibility of samples affected by NIHL due to occupational cause, and even if there was NIHL present in some cases, the group exposed to COVID-19 still presented worse tonal thresholds. The presence or absence of audiovestibular symptoms and other comorbidities was left to chance in this group, to ensure the representation of the general population.

In the present study, both groups were evaluated during the same period (2021–2023). This is because there is heterogeneity of the previous results in relation to the occurrence of hearing loss during and before the pandemic in question. While some research shows an increase in the incidence of hearing loss during the COVID-19 pandemic, others have shown its decrease compared to the pre-pandemic period, probably due to the fear of patients becoming infected with Sars-Cov-2 when seeking health care in the presence for this symptom. 10 14

In the exposed group, females predominated (75.8% vs. 24.2% males), likely reflecting the lower rate of health-seeking behavior among menin Brazil, 18 in contrast to the unexposed group, where sex distribution was equal (50% each).

The unexposed group comprised individuals who underwent mandatory occupational audiometry regardless of health-seeking behavior. The sex imbalance between groups was due to the random nature of participant selection and does not compromise the primary objective of the study — to compare audiometric thresholds according to COVID-19 exposure status.

The medians of the ages of the two groups were close, with a clinically non-significant difference (34.5 years for the exposed group and 32 years for non-exposedgroup), avoiding the results suffering from the influence of age extremes.

Only two participants required ICU hospitalization during COVID-19 infection, and both presented normal pure-tone thresholds, thereby minimizing potential confounding from ototoxicity and other hearing-damaging factors common in ICU settings. Although ototoxic medication use was not matched between groups, this potential confounding factor was partially mitigated by the exclusion of individuals with hearing-affecting comorbidities (and their associated ototoxic treatments) in the exposed group. In the unexposed group, comorbidities occurred at random, reflecting the general population. Despite these differences, the exposed group still exhibited worse tonal audiometric thresholds, supporting the association between COVID-19 exposure and hearing alterations.

The statistical results of the present study showed that the medians of the thresholds for each hearing frequency studied were predominantly worse in exposed individuals than in non-exposed individuals in both ears (as shown in Fig. 1 ). Additionally, the hearing loss rate was higher in the exposed group (28.3%) compared to the non-exposed group (15%). The results also demonstrated that the likelihood of alteration in tonal thresholds is approximately twice as high in patients exposed to COVID-19 compared to those not exposed (OR 2.24 [95% CI 1.18 to 4.25]).

The right and left ears of each exposed individual, when compared, revealed different behavior between them after Sars-Cov-2 contamination. The 500 Hz frequency had higher thresholds in the right ears and the 4000, 6000 and 8000Hz frequencies had higher thresholds in the left ears. The frequencies of 250, 1000, 2000 and 3000Hz showed no statistically significant difference between the ears. This result allows us to infer that the hearing loss induced by Sars-Cov-2 can asymmetrically affect the ears of an individual.

Only SNHL was identified in the exposed group, whether unilateral or bilateral. This finding is consistent with the review by Mehraeen et al. (2023), which reported a predominance of SNHL cases, with rare reports of conductive or mixed hearing loss, likely attributable to pre-existing conditions or methodological limitations in the reviewed studies. In another systematic review assessing the impact of COVID-19 on the incidence of sudden deafness, unilateral and bilateral hearing loss were also found. 10

The persistence of audiometric changes in individuals exposed to COVID-19 for more than six months after the diagnosis of the disease (62.5% of the total sample) allows us to infer that Sars-Cov-2 infection can generate hearing sequelae. Neither Ong & Cruz (2021), in their scoping review, nor Frosolini et al. (2022), in their systematic review and meta-analysis, had definitively answered this question. 14 15 We believe that further studies aimed at evaluating the prevalence of acute or persistent hearing loss are necessary.

The predominant ENT symptoms in COVID-19 in the present study were nasal symptoms (67.5%) and cough (65.8%), followed in descending order by loss of olfaction (60%), loss of taste (56.7%) and sore throat (47.5%) and, less frequently, dizziness (30.8%) was the most common audiovestibular symptom, followed by tinnitus (19.2%), ear pain (18.3%), hearing loss (18.3%) and only one case of peripheral facial paralysis (0.8%). Hoarseness (21.7%) was also observed among the participants.

The frequencies of ENT symptoms caused by COVID-19 found in the literature are similar to our results; with a predominance of cough, sore throat, nasal symptoms, dysgeusia and hyposmia and, less frequently, the audiovestibular symptoms; however, these generate greater concerns and make necessary the proper management of affected patients. 16 17 18 19 Although audiovestibular symptoms are not the most common ofthe ENT symptoms, they are present with frequencies that are sufficiently high enough to be part of the clinical conditions for the diagnosis of COVID-19.

Individuals infected with SARS-CoV-2 who reported hearing loss had a significantly higher odds of audiometric threshold alteration (OR = 8.9; 95% CI: 3.2–24.9). It is a symptom that cannot be neglected and should always be investigated with audiometry and, if necessary, early treatment to avoid hearing sequelae.

Participants who complained of tinnitus during COVID-19infection in our study were 3.7 times more likely to have tonal threshold changes than the other participants (OR = 3.7 [95% CI 1.4; 9.6]). Tinnitus is one of the most common symptoms associated with sudden sensorineural hearing loss in COVID-19, according to Meng et al. (2022). 10 This symptom can be a signal of hearing loss due to COVID-19, and should always be investigated with audiometry.

In our sample, no patient experienced hearing loss or tinnitus alone, but always accompanied by other ENT symptoms. Although there are limited reports of hypoacusia as a sign of the onset of the disease, mostly it is accompanied by other symptoms. 10 20 Only one participant showed dizziness as a single symptom of COVID-19. Although dizziness is most frequently presented among the audiovestibular symptoms caused by COVID-19 in this study, we found no statistically significant association between it and hearing loss.

Altered hearing thresholds were identified in 19 participants who did not report hearing loss during COVID-19, possibly due to subclinical loss during the acute phase, pre-existing undetected loss, or recall bias. Seven participants who were positive for Sars-Cov-2 reported hearing loss, but no changes in tonal thresholds were observed, so it was probably a transient symptom or a bias of response.

There is a possibility that asymptomatic SARS-CoV-2-infected individuals are present in the unexposed group, which could overestimate the prevalence of hearing impairment in that group. However, as the results showed that the exposure group was the most affected, this was not a limiting factor for the study, but a reinforcement for our hypothesis.

This study presents some methodological limitations that must be considered when interpreting its findings. The exposed group comprised patients attending a general ENT outpatient clinic, constituting a convenience sample and may not accurately represent the general population of individuals infected with SARS-CoV-2. While the not exposed group was obtained from an occupational health clinic, this difference in recruitment context results in distinct population profiles, introducing a potential selection bias.

## Conclusion

In the present study, individuals exposed to COVID-19 demonstrated worse pure-tone thresholds and a higher rate of hearing threshold alteration compared with unexposed individuals. These findings suggest that COVID-19 may be associated with hearing loss. Additionally, a statistically significant association was identified between audiometric threshold alterations and self-reported hearing loss and tinnitus during COVID-19.
